# The Hypolipemic Properties of Kaempferol Presented in Microwaved Cooked Broccoli Between Hyperlipidemic Rat Models

**DOI:** 10.1002/fsn3.70556

**Published:** 2025-07-07

**Authors:** Asmahan A. Ali, Huda Aljumayi, Thamer Aljutaily, Hani A. Alfheeaid, Nada Bint Abdullah Al‐Zunaidy, Isam A. Mohamed Ahmed, Belal M. Mohammed, Nazeha A. Khalil

**Affiliations:** ^1^ Department of Food Science and Human Nutrition, College of Agriculture and Food Qassim University Buraydah Saudi Arabia; ^2^ Department of Food Science and Nutrition, College of Sciences Taif University Taif Saudi Arabia; ^3^ Department of Food Science and Nutrition, College of Food and Agricultural Sciences King Saud University Riyadh Saudi Arabia; ^4^ Department of Food Science and Technology, Faculty of Agriculture and Food Sciences Ibb University Ibb Yemen; ^5^ Nutrition and Food Sciences Department, Faculty of Home Economics Menoufia University Shebin El Kom Egypt

**Keywords:** antioxidant, cholesterol, flavonoid and plant‐based diets, triglyceride

## Abstract

Kaempferol is a natural flavonoid abundant in many plant‐based foods; eatable fruits and vegetables including broccoli. It has been used for its potential health benefits, including hypolipidemic properties and a wide range of pharmacological activities as antioxidants contributing to numerous diseases treatment. The current study aimed to measure kaempferol between microwaved broccoli (MB) samples using high‐performance liquid chromatography (HPLC). Also, their potential hypolipidemic properties between hyperlipidemic rats were investigated: MB at different times (1, 2 and 5 min) fed randomly rat groups by 2.5% each with two control groups positive vs. negative, and collected blood samples were used for blood glucose (BG) and lipid profiles cholesterol; CHO, triglycerides; TG, high‐density lipoprotein‐cholesterol (HDL‐c), and low‐density lipoprotein‐cholesterol (LDL‐c) and again for kidney (Urea, Criatinine) and liver (alanine & aspartate aminotransferase) functions with histological analysis. Collected data showed kaempferol levels only in two MB samples (2 & 5 min; about 56 and 68 mg/100 g, respectively). While the animals' body weight levels declined with different improvements in lipids, kidney, and liver functions. The lowest BG was seen with rats fed a 2 min microwaved cooking period (104.33 ± 1.15 mg/dL) that was close to control rats (102.67 ± 2.08 mg/dL). Also, HDL‐c increased and LDL‐c decreased while the biggest shown within rats fed 1 min samples (33.80 ± 2.83 mg/dL). Overall, kaempferol in MB demonstrated potential in modulating levels of BG, lipids, kidney, and liver functions between hyperlipidemic models; thus, incorporating MB at 2–5 min influenced the antioxidant levels, providing a valuable adjunct to traditional lowering lipid therapy.

## Graphical abstract

The current study aimed to measure Kaempferol between microwaved broccoli (MB) samples using High performance liquid chromatography (HPLC). Also, their potential hypo‐lipid properties between hyper‐lipid rats were investigated. Kaempferol in microwaved broccoli demonstrated potential in modulating levels of BG, lipids, kidney and liver functions between hyper‐lipid models thus incorporating microwaved broccoli at 2‐5 min influenced the antioxidant levels providing a valuable adjunct as traditional lowering lipid therapy.

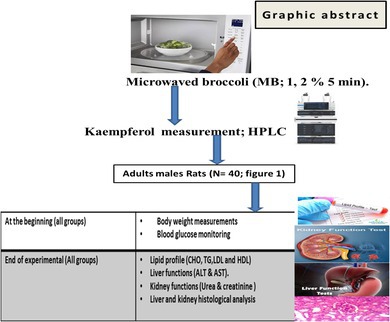

## 1 Introduction

The antioxidant activities are widely known with their protective effects in many different diseases, especially cardiovascular diseases (CVDs) and their risk contributor such as stroke, hyperlipidemia, diabetes, and inflammatory bowel diseases (Hijová 2023; Aljumayi et al. 2024). Kaempferol, identified also as kaempferol‐3 or kaempferide which is a natural flavonoid presented in tea and many common plant‐based foods mainly leafy green vegetable and fruits such as red onions, tomatoes, broccoli sprouts, kale and spinach, broccoli, cabbage, gooseberries, grapes, strawberries, citrus fruits and grapefruit (Ren et al. 2019; Calderón‐Montaño et al. 2011). Kaempferol is an antioxidant that is well known for its potential health benefits, including hypolipidemic properties and many different benefits in treating numerous diseases by its wide range of pharmacological activities, including antioxidant, anti‐inflammatory, antimicrobial, anticancer, neuroprotective and antidiabetic, and anti‐osteoporotic (Calderón‐Montaño et al. 2011; Aljumayi et al. 2024).

The World Health Organization (WHO) indicated that CVDs cause deaths by about 17.9 million people every year while it has estimated that CVDs prevention and treatment in the next 25 years could cost up to $47 trillion (Behl et al. 2020).

Some epidemiological studies show a positive correlation between the intakes of rich dietary kaempferol levels and risk factor reduction of developing several disorders, e.g., cancer and CVDs, in addition to atherosclerosis, coronary heart disease, hyperlipidemia, obesity, and diabetes (Yao et al. 2024). Also, it has been shown to improve cardiac functions and decrease apoptosis, as kaempferol's effects on cardiac hypertrophy were demonstrated to evaluate cardiac function in vivo and in vitro in studies that were treated with/without kaempferol (100 mg/kg/d). It has been revealed significantly that kaempferol could protect the mouse heart and HT29 cells from pathological oxidative stress; models treated with kaempferol were protected against cardiac hypertrophy and contributed to overall cardiovascular health (Feng et al. 2017; Calderón‐Montaño et al. 2011). That may help decrease lipid profile levels, particularly cholesterol levels. Therefore, CVDs have been associated with many factors, such as food intake and the cooking methods used followed by dietary patterns. For example the traditional Western dietary pattern exacerbates the elevation of CVDs and their risk problems. Such dietary patterns are well identified by high levels of animal products and processed foods, while they are low in fruits and vegetables, in addition to the levels of whole grains, which in turn can increase the risk levels of CVDs (Johnson et al. 2019; Dabeek and Marra 2019). On the other hand, plant dietary sources rich in vegetables, such as broccoli ( *Brassica oleracea* ), which is a cruciferous vegetable, like kale, cauliflower, Brussels sprouts, and cabbage, are important for reducing many human health problems, especially CVDs and their effects. Such vegetables contain many antioxidants that are associated with potential hypolipidemic effects, supporting many aspects of beneficial healthy activities such as kaempferol and other phytochemicals.

Broccoli is low in calories but contains many different nutrients and antioxidants, including fiber, vitamins (Vitamin C, K & E), and minerals (calcium, potassium, potassium) that support many aspects of beneficial healthy activities (U.S. Department of Agriculture 2019). Additionally, it has many different natural plant components like antioxidants and carotenoids that can prevent and lower the chances of having heart disease in addition to boosting the immune system (Lin and Chang 2005). Again, a sulfur compound called sulforaphane (a sulfur‐containing compound that gives cruciferous vegetables their bitter bite) has shown to help certain health conditions such as CVDs, obesity, and diabetes. It also might stop cancer cells from forming in the human body, so it is good for broccoli to be used as a natural dietary fiber and antioxidant source. Most important to our current study is the kaempferol, which has correlated well with many human health conditions that may help in lipid management (Dabeek and Marra 2019). However, such nutrient levels depend on many factors such as the parts eaten (florets, leaves, stems) and preparation or cooking methods. Each method has its own advantages and disadvantages; e.g., boiling will remove up to 90% of broccoli's nutrients, so it is recommended to prepare it in a different way, such as roasting, steaming, stir‐frying, or microwaving (SR Legacy Food Category 2019). Also, uncooked vegetables were reported previously to bind the bile acid that has been associated with cholesterol levels. Additionally, the levels of kaempferol may be affected by the cooking methods due to the heat sensitivity when broccoli is cooked. However, such affected presence levels of kaempferol have not been measured within the MB in association with their beneficial properties on hyperlipidemia, which requires further research. The cooking methods can alter the recirculation of bile acids in order to affect cholesterol utilization and fat absorption (Kahlon et al. 2008). Also, it has been reported that the steam cooking method significantly enhanced the in vitro bile acid binding of broccoli compared with bile acid binding values observed with uncooked samples (Kahlon et al. 2008; Xianli et al. 2019). Many vegetables in our daily diet are regularly steam‐cooked, such as steam‐cooked greens, kale, broccoli, and cabbage, for many health‐promoting benefits, especially lowering the risk of cardiovascular disease and cancer. Including and incorporating plant‐based foods such as cooked broccoli along with a healthy balanced diet and additionally healthy lifestyle habits would greatly help contribute to overall health and potentially support lipid management (Aljumayi et al. 2024).

So the current study aimed to evaluate the levels of kaempferol present in samples of different MB, and cooking methods affecting the integrity and concentration of kaempferol in broccoli and its subsequent impact on lipid metabolism. Additionally, the effects of MB samples (different time points; 1, 2 and 5 min) on the health states were determined between hyperlipidemic animal models, evaluating their hypolipidemic properties compared to the control healthy group. Also, animals' body weights were recorded and calculated, and then collected serum blood samples were kept at −80°C until further analysis (glucose, lipid profiles, kidney and liver functions) in addition to kidney and liver histological analysis.

## 2 Materials and Methods

### 2.1 Foods and Chemicals

Green fresh broccoli (*Brassica oleracea*) samples were obtained from the local market (Shibin El‐Kom, Menoufia, Egypt) and stored at 4°C, then used within 24 h for the following cooking and used procedures. Kaempferol analytical standards were obtained from Extra Synthese (Genay, France). However, hydrochloric acid (HCL) and methanol (MEOH; HPLC grade) were purchased from Sigma‐Aldrich (Germany). Kaempferol stock solution was prepared in dimethyl‐sulfoxide (DMSO) and stored at −20°C.

### 2.2 Sample Preparation

All stored broccoli samples were washed under running water tap, cut into slight pieces, and mixed randomly before being cooked; microwaved in water (10 g + 300 mL H_2_O) for 3 different time points (1, 2 and 5 min; *n* = 3 each) prior to the Kaempferol extraction process according to Reeves et al. (1993) and Khalil (2017) with some modifications. The selected cooking parameters were modified based on collective data from the literature (Christensen 2016; Ramsey 2017; Roskelley 2018).

### 2.3 Extraction Process

Fresh raw and microwaved cooked broccoli samples (10 g) were added to 15 mL of 80% MEOH, then they were homogenized as described before by Khalil (2017). The mixtures were filtered via four layers of cheesecloth, and the residue (1 g) was homogenized again with 62.5% MEOH (40 mL) for 20 min in ultrasonic. For hydrolysis conditions, HCl (2 mL, 2 M) was added and heated again for 30 min at 90°C. Finally, samples in total were made up with 50 mL MEOH after being cooled down to room temperature and before being sonicated for 5 min. All the filtered (0.002 μL filters) collected supernatants were stored at 4°C in a refrigerator before being used for HPLC analysis.

### 2.4 Kaempferol Determination by HPLC

Stock solution of kaempferol was prepared for kaempferol identifications, dissolving 100% MEOH in an appropriate amount, then its levels were determined. All the clear broccoli extract supernatants (100 μL) were analyzed under chromatographic conditions with a flow rate of 1 mL·min^−1^ and the column temperature set at 25°C (Agilent Technologies 1200 Series HPLC equipped were used with a diode array detector DAD G1315B, Zorbax Eclipse XDB‐C18 column; 4.6 × 150 mm, 5 μm particle size, Agilent, Palo Alto, CA, USA). Again, 20 μL was used as the injected volume and samples were detected at 350 nm. The separated peaks were identified by comparing with the retention time of individual standard peaks as previously described by our previously published data (Khalil 2017).

### 2.5 Experimental Animal Model

**FIGURE 1:**
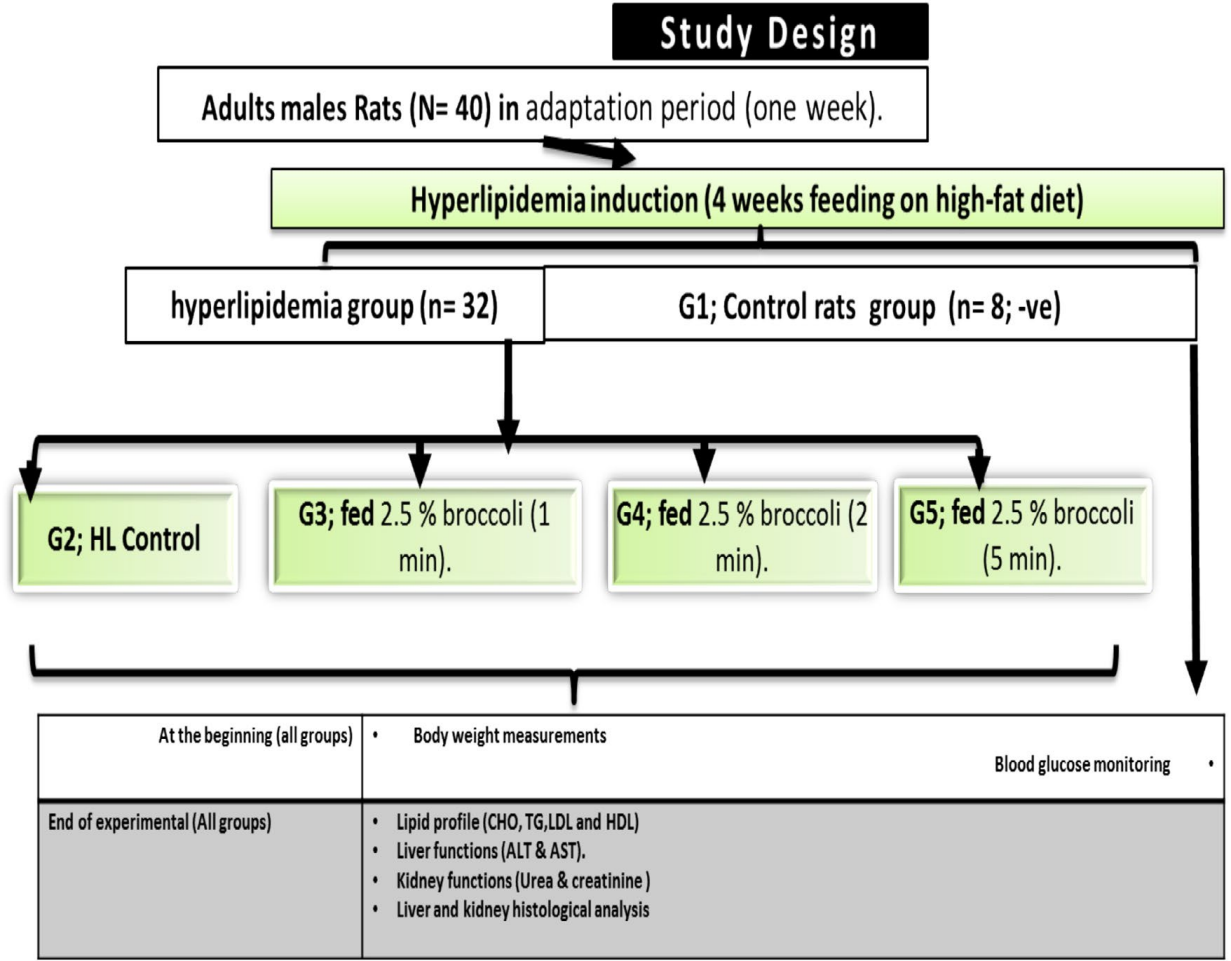
The current study design between used animal models.

Forty male albino rats aged 6 weeks and weighing 110–120 g were obtained from the National Training Center, Cairo, Experimental Animal Care Centre, Egypt. All rats were fed a control normal diet for an adaptation period (1 week; 10% kcal from fat). The control normal diet was formulated as in the supplemental material and described by Aljutaily et al. (2022) and Khalil (2017), as well as in the supplemental table (Table S1) for rodent growth according to the American Institute of Nutrition recommendation (Reeves et al. 1993). All rats were then divided randomly into five groups (*n* = 8 rats each) as described in Table 1; four animal groups were fed high‐fat diets for 6 weeks (hyperlipidimia induction; HFD) as described by our previously published studies Aljutaily et al. (2022) and Li et al. (2022) that contained 60% kcal from fat. One group was fed a control normal diet to be used as a negative control healthy group (G1). Three hyperlipidemia groups out of the four received water and diet for up to 4 weeks with 2.5% broccoli addition each but at three different microwaved cooking time points (1, 2 and 5 min; G3:G5) while the fourth was used as the control (+) hyperlipidemia group (G2) as described in Figure 1. The supplemented diets of all rat models were specified according to Table 1, with different MB cooking time points added to the basal dietary meal. All animal body weights were recorded before and after running the experiment. The gained body weight was calculated by comparing it to the control positive hyperlipidemia group (group body weight − control positive body weight). Blood samples collected at the end of the experiment were kept at room temperature for 30 min and then centrifuged at 3500 rpm for 15 min to collect the serum samples, which were kept at −80°C until further biochemical analysis.

**TABLE 1:** Basal dietary meal of rat groups used within the current experiment.

Group code	Group description
Control (−ve)	Healthy normal rats fed basal diet
Control (+ve)	Hyperlipidemic rats fed basal diets
MB; 1 min	Hyperlipidemic rats fed basal diet supplemented with broccoli microwaved for 1 min
MB; 2 min	Hyperlipidemic rats fed basal diet supplemented with broccoli microwaved for 2 min
MB; 5 min	Hyperlipidemic rats fed basal diet supplemented with broccoli microwaved for 5 min

### 2.6 Biochemical Analysis

Most of the current biochemical analysis methods were determined with little adjustment to the method, followed by our published data (Khalil et al. 2021; Aljutaily et al. 2022). Serum blood glucose (BG), lipid profile, kidney, and liver functions were determined within the following analytical procedures as described according to the method of Lai and Chen (2024) and that was also followed for serum glucose and serum total cholesterol (CHO). Once more, serum triglyceride (TG) was determined by enzymatic method using kits according to Xu et al. (2024) although high‐density lipoprotein‐cholesterol (HDL‐c) was established additionally, low‐density lipoprotein‐cholesterol (LDL‐c) according to the method described by Martins et al. (2022). Regarding the liver functions, ALT (alanine aminotransferase) and AST (aspartate aminotransferase) determinations were carried out according to Draz et al. (2020) whereas urea with serum creatinine was determined according to the method described by Shekoufeh et al. (2023).

### 2.7 Histology Structure Examinations

This study used the collected experimental organ samples, especially (kidneys and livers) for histological structure evaluations. All collected organ samples were fixed in neutral formalin buffered (at 10%) and after being dehydrated in alcohol and cleared in xylol were processed routinely and embedded in wax paraffin. Finally, all prepared paraffin blocks sectioned (at 4–5 μm thickness) and also as described within our previously published data, stained with Hematoxylin and Eosin (Khalil et al. 2021).

### 2.8 Statistical Analysis

The data are expressed as the mean ± standard deviation (SD). The statistical analysis was carried out using SPSS version 21.0 software (SPSS Inc., Chicago, IL, USA), while the differences between the groups were analyzed using one‐way analysis of variance (ANOVA). Also, differences between pairs of means were subsequently tested using Duncan's multiple range as a post hoc test. Data were considered statistically significant differences at *p* ≤ 0.05.

## 3 Results

### 3.1 Kaempferol Measured by HPLC

The stander kaempferol solution (10 μM) at time (min) and wavelength 350 nm has been used within HPLC results in order to present the obtained data for our examined samples and after the calibration process in Table 2. As it can be seen in Table 2 that all the microwaved samples have not got any kaempferol levels, which means no detection (ND) in the following table (Table 2).

**TABLE 2:** Kaempferol levels measured by HPLC of microwaved broccoli at different time points (1, 2 and 5 min).

Treatments	Kaempferol levels mg/100 g
Fresh broccoli	ND
Microwaved Broccoli; 1 min	ND
Microwaved Broccoli; 2 min	56
Microwaved Broccoli; 5 min	68

*Note:* Data represent mean ± SD (*n* = 3).

Abbreviation: ND = no detection.

It can be seen from Table 2 that kaempferol was at the biggest levels in broccoli samples microwaved at 5 min, which was followed by the 2 min cooking period (68 and 56 mg/100 g, respectively). However, samples either microwaved for 1 min and/or fresh broccoli samples did not present any kaempferol levels (ND) so the cooking methods and times used have affected the kaempferol release levels within our tasted broccoli samples.

### 3.2 The Effect of Cooked Broccoli Samples on Body Weight Gain Between Hyperlipidemic Animal Models

Animal models in the current study have been used to measure the effects of different MB consumptions on their body weight gain (BWG) level at different time points (1, 2, and 5 microwaved min). Table 3 illustrates the collected data and shows that all the animal models used had similar body weight levels (BW; about 120 g). However, at the end of running the experiment, all the groups achieved different BW levels; all have increased. The biggest BWG levels were obtained with the control group (364.66 ± 2.51 g), which was followed by the rats fed cooked MB samples with a 1 min cooking period. Again, this group was followed by the rats consuming cooked MB samples with a 5 min cooking period (344.00 ± 3.60 g). While the final animal groups were those fed a normal basal diet and cooked MB samples with a 2 min cooking period, they were at similar levels (331.33 ± 3.21 and 331.33 ± 1.00, respectively; Table 3).

**TABLE 3:** Effect of microwaved broccoli at different time points on body weight gain between used rat groups.

Groups	Initial body weight (g)	Final body weight (g)	Change of BWG from control (+; g)
Control (−ve); healthy	119.67 ± 1.52^a^	331.33 ± 3.21^a^	−33.33
Control (+ve) unhealthy	118.67 ± 1.52^a^	364.66 ± 2.51^d^	—
MB; 1 min	118.67 ± 1.15^a^	351.00 ± 1.73^c^	−13.66
MB; 2 min	119.33 ± 1.52^a^	331.33 ± 1.00^a^	−33.33
MB; 5 min	120.67 ± 2.64^a^	344.00 ± 3.60^b^	−20.66

*Note:* Data represent mean ± SD (*n* = 8). Means in the same column with different superscript letter are significantly different (*p* ≤ 0.05). MB means microwaved broccoli samples cooked for different time periods and FBW means final body weight.

Regarding the changes of BWG from the control positive rats group, Table 3 illustrates that both animal groups fed MB samples at the 2 min time point and the negative control group got similar effects on the rats' BW (decreased by about 33 g). Again, rats that consumed 5 min MB presented a reduction in their FBW by about 20 g, while the group of rats fed 1 min cooked broccoli showed the lowest declined levels (about 14 g).

### 3.3 The Effect of Cooked Broccoli Samples on Serum Glucose Levels Between Hyperlipidemic Animal Models

The collected serum glucose levels obtained from the hyperlipidemic rats are shown in Table 4. It can be noticed that all the rat groups had low serum glucose levels significantly (*p ≤ 0.05*) except the group fed cooked MB samples with a 1 min microwaved period (142.33 ± 3.78 mg/dL). The lowest levels were seen with the animal model fed a 2 min microwaved period (104.33 ± 1.15 mg/dL), which was close to the rats fed a basal control diet (102.67 ± 2.08 mg/dL). The second lowest rat group with serum glucose levels was the animal models that consumed broccoli at a 5 min microwaved cooking period (119.00 ± 2.64 mg/dL). Finally, the largest serum glucose levels were obtained with the group served broccoli at a 1 min microwaved period (142.33 ± 3.78 mg/dL). Relative changes in BG levels between all groups to the positive control group; it can be noticed from Table 4 that glucose levels have declined among all the animal models used in the current experiment compared to the positive group levels, which had the highest glucose level among all the groups (158.67 ± 1.52 mg/dL). The rats fed broccoli samples microwaved for 2 min showed the lowest glucose levels significantly (decreased from the positive group by −54.34 mg/dL) compared to all the hyperlipidemia groups. This group was followed by the reduction in glucose levels of the animal model that consumed 5 min cooked broccoli samples and finally by the 1 min cooked samples (39.67 and 16.34 mg/dL reduction levels respectively).

**TABLE 4:** Effect of microwaved broccoli at different time points on blood glucose levels between hyperlipidemic rats.

Groups	Glucose level (mg/dL)	Relative change of control (+; mg/dL)
Control (−ve); healthy	102.67 ± 2.08^a^	−56
Control (+ve); unhealthy	158.67 ± 1.52^d^	—
MB; 1 min	142.33 ± 3.78^c^	−16.34
MB; 2 min	104.33 ± 1.15^a^	−54.34
MB; 5 min	119.00 ± 2.64^b^	−39.67

*Note:* Data represent mean ± SD (*n* = 8). Means in the same column with different superscript letters are significantly different at (*p* ≤ 0.05). MB meanS microwaved broccoli samples cooked for different time periods.

### 3.4 The Effect of Cooked Broccoli Samples on Lipids Profile Levels Between Hyperlipidemic Animal Models

**FIGURE 2:**
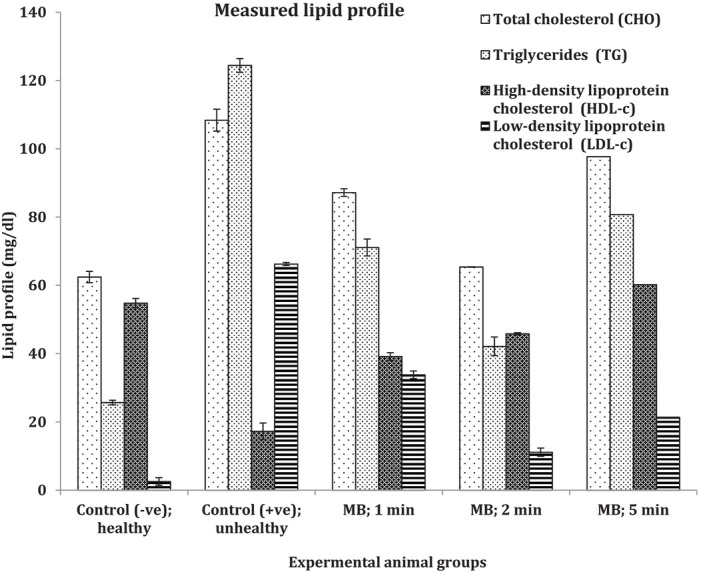
Measured lipid profile.

The presented data in Table 5 and Figure 2 revealed the measured lipid profile levels (CHO, TG, HDL, and LDL) between the used animal models. It can be noticed that the highest significant cholesterol (CHO) levels were seen with rat group that consumed MB samples cooked for 5 min (97.70 ± 1.10 mg/dL). It has been followed significantly by the rat group that consumed microwaved cooked samples for 1 and 2 min (87.17 ± 1.96 and 65.37 ± 1.40 mg/dL, respectively), which was so close to the control negative group (fed basal diet; 62.43 ± 1.65 mg/dL). Regarding the triglyceride levels; Table 5 and Figure 2 present that the largest significant triglyceride levels were seen within rats group fed 5 min MB samples (97.7 ± 1.10 mg/dL), and that was followed by group fed 1 min cooked broccoli samples (87.17 ± 1.96 mg/dL). Finally the rats fed with samples of 2 min cooking time significantly reached 65.37 ± 1.40 mg/dL.

**TABLE 5:** Effect of microwaved broccoli at different time points on lipid profile between hyperlipidemic rats.

Lipid profile (mg/dL)				
Groups	Total cholesterol (CHO)	Triglycerides (TG)	High‐density lipoprotein‐cholesterol (HDL‐c)	Low‐density lipoprotein‐cholesterol (LDL‐c)
Control (−ve); healthy	62.43 ± 1.65^a^	25.67 ± 3.21 ^a^	54.77 ± 1.11^d^	2.53 ± 0.049^a^
Control (+ve); unhealthy	108.37 ± 0.66^e^	124.40 ± 2.02^e^	17.25 ± 2.48^a^	66.23 ± 2.75^e^
MB; 1 min	87.17 ± 1.96^c^	71.07 ± 1.10^c^	39.15 ± 3.40^b^	33.80 ± 2.83^d^
MB; 2 min	65.37 ± 1.40^b^	42.13 ± 2.41^b^	45.81 ± 1.12^c^	11.13 ± 0.30^b^
MB; 5 min	97.70 ± 1.10^d^	80.73 ± 0.47^d^	60.19 ± 1.12^e^	21.37 ± 1.20^c^

*Note:* Data represent mean ± SD; values not sharing superscript letters in the same column are significantly different (*p* ≤ 0.05). MB means microwaved broccoli samples cooked for different time periods.

All collected data are in comparison between unhealthy and healthy control groups significantly; they were about 108.37 ± 0.66 and 62.43 ± 1.65 mg/dL, respectively. Additionally, HDL‐c levels represented the highest levels significantly in groups fed 5 min MB samples (60.19 ± 1.12 mg/dL) followed by 2 min cooked MB samples (45.81 ± 1.12 mg/dL) and finally was the 1 min broccoli cooked samples (15 ± 3.40 mg/dL) comparing to the control positive group that was closer significantly to the group fed 5 min MB samples (54.77 ± 1.11 mg/dL) while the positive control group was with the lowest level at 17.25 ± 2.48 mg/dL.

LDL‐c shown in Table 5 and Figure 2 were with its biggest significant levels within the animal model that got the cooked broccoli samples at the 1 min time point (33.80 ± 2.83 mg/dL), and that was the closest to the positive control group (66.23 ± 2.75 mg/dL). The following groups were within the rats that consumed broccoli samples at the 5 min cooking time (21.37 ± 1.20 mg/dL) then the rats fed 2 min cooking broccoli samples (11.13 ± 0.30 mg/dL) while the control negative group was significantly the lowest among all the used animal models with about 3 mg/dL only. To conclude all levels of the collected lipid profiles are in good arrangements and correlated well with the measured kaempferol levels as the measured antioxidant presented early in the broccoli microwaved samples (Table 2; Figure 2). Lipid profile improved among the used hyperlipidemia animal models, especially between rats fed 2 min MB samples.

### 3.5 The Effect of Cooked Broccoli Samples on Kidney and Liver Functions Between Hyperlipidemic Animal Models

Values of biochemical parameters: kidney and liver functions are presented in Table 6 again (urea and creatinine in addition to ALT and AST levels). The table illustrates the effects of used MB samples on kidney and liver functions between used animal models. It can be noticed that kidney function, especially urea levels, were at the highest levels between the rats group that consumed 5 min MB samples, which were 35.50 ± 1.50 mg/dL. While the lowest levels were seen between the rats group fed 2 min MB samples (about 29.66 ± 1.52 mg/dL) among the animal models used in the current experiment, compared to healthy and unhealthy control groups that were approximately 24.0 ± 3.60 mg/dL and 42.33 ± 2.51 mg/dL, respectively.

**TABLE 6:** Effect of microwaved broccoli at different time points on kidney and liver functions between hyperlipidemic rats.

Groups	Kidney functions		Liver functions	
	Urea (mg/dL)	Creatinine (mg/dL)	ALT (U/L)	AST (U/L)
Control (−ve); healthy	24.0 ± 3.60^a^	0.49 ± 0.02^a^	26.10 ± 1.2^a^	49.0 ± 1.0^a^
Control (+ve); unhealthy	42.33 ± 2.51^e^	1.23 ± 0.02^c^	49.60 ± 12.0^c^	137.67 ± 1.5^e^
MB; 1 min	32.33 ± 3.21^cd^	0.67 ± 0.16^b^	49.43 ± 1.2^c^	80.0 ± 1.0^c^
MB; 2 min	29.66 ± 1.52^bc^	0.51 ± 0.02^a^	32.07 ± 1.1^ab^	66.67 ± 1.15^b^
MB; 5 min	35.50 ± 1.50^d^	0.64 ± 0.02^b^	38.00 ± 2.0^b^	110.67 ± 2.08^d^

*Note:* Data represent mean ± SD; values not sharing superscript letters in the same column are significantly different (*p* ≤ 0.05). MB means microwaved broccoli samples cooked for different time periods.

Additionally, the liver functions in Table 6 also shown that ALT where at its peak levels between groups fed 1 and 5 min (49.43 ± 1.2 and 38.00 ± 2.0 U/L) broccoli samples compared to the lowest levels of group feed broccoli samples at 2 min (32.07 ± 1.1 U/L). Again levels of AST at 5 min microwaved time point were about 110 U/L compared to the lowest levels between rats fed 1 min of cooked broccoli samples that were around 80 U/L comparing to the positive and negative groups (137.67 ± 1.5 and 49.0 ± 1.0 U/L, respectively).

### 3.6 The Effect of Cooked Broccoli Samples on Kidney and Liver Histological Analysis Between Hyperlipidemia Animal Models

**FIGURE 3:**
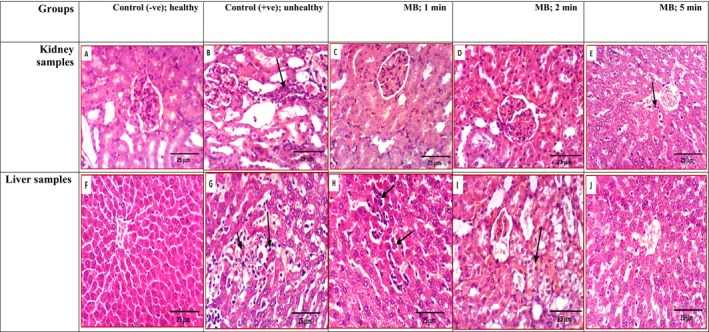
The kidney obtained from different rats as (A) control healthy group, while (B) is a section of control hyperlipidemic rats and (C) is the kidney of rats fed MB cooked for 1 min. Kidney sections presented at (D and E) represented rats from the group fed MB cooked for 2 and 5 min respectively (H & E × 400). Again Figure 1: The examined liver samples demonstrated control healthy rats presented in (F) and the liver of a rat from control hyperlipidemic shown in (G) while the liver of rats fed MB cooked for 1 min (H). Finally, Figure 3 of I and J illustrated the liver of a rat fed MB cooked for 2 and 5 min (H & E × 400) respectively. MB, microwaved broccoli.

Histological examination of both kidney and liver sections has been demonstrated in Figure 3, which focused on evaluating renal and liver function and structure, presenting the kidney and liver sections among all groups of rats used The kidneys of rats from the control group (healthy; −ve) exhibited the normal histological structure of renal parenchyma (H & E × 400) as presented in Figure 3A. Figure 3B illustrates the kidney of rats from the control hyperlipidemic group 2 (unhealthy; +ve), showing interstitial nephritis (H & E × 400). Additionally, the same Figure 3D,E shows the kidneys of rats from other treatments fed MB cooked for 2 and 5 min with no histopathological changes (H & E × 400) but Figure 3C of kidneys from rats fed MB for 1 min shows vacuolation of epithelial lining of renal tubules (H & E × 400).

The liver collected samples have been used for histological analysis, illustrated in Figure 3F–J as (F) showing the control healthy group, with a normal histological structure of the hepatic lobule (H & E × 400) and (G) from the hyperlipidemia control group showing focal hepatocellular necrosis and apoptosis associated with mononuclear inflammatory cells infiltration (H & E × 400). Liver samples of rats fed MB for 1 min are presented in (H), showing focal hepatocellular necrosis associated with mononuclear inflammatory cells infiltration (H & E × 400). Finally, Figure 3 at (I) and (J) illustrated the liver of a rat fed MB cooked for 2 and 5 min, and showing slight cytoplasmic vacuolization of hepatocytes (H & E × 400) in addition to no histopathological changes (H & E × 400) respectively.

## 4 Discussion

The measured kaempferol, as a bioactive compound found in broccoli, mainly in the current study, is to mitigate hyperlipidemia. The current study aimed to assess the hypo‐lipid properties of kaempferol and shed light on potential mechanisms involved in lipid regulation. Collected data presented that kaempferol was at the highest levels in broccoli samples microwaved at 5 min and that was followed by the 2 min cooking period (68 and 56 mg/100 g, respectively). Both the cooking methods and times used in this study have affected the kaempferol released levels. Indeed, such collected data are in agreement with previously published data that show that different food preparation or cooking procedures and time used strongly could affect the availability of bioactive and antioxidant compounds, especially the heat treatment (Khalil 2017; Yu et al. 2018). Additionally, the levels of changed BWG illustrate that both animal groups fed MB samples at 2 min and negative control group have similar effects (decreased by about 33 g). While the rats consumed 5 min MB presented FBW reduction by about 20 g and the group rats fed 1min cooked broccoli showed the lowest declined levels (about 14 g). Definitely the current collected data presented herein are highly supported with different previous similar effects especially in correlation to the kaempferol levels revealed with the timed cooking conditions. For example, rats fed high‐fat diets with boiled broccoli samples showed reduction in their BW (Khalil 2017). Again mice fed hypercaloric diet in addition to kaempferol consumption had BW reduction in respect to the controls positive group that only fed hypercaloric diet (Zang et al. 2015).

Glucose levels have declined between all the animal models used in the current experiment compared to the positive group levels that had the highest glucose level. The lowest glucose levels were in rats fed broccoli samples microwaved for 2 min compared to all the hyperlipidemia groups, followed by the reduced glucose levels of the animal model that consumed 5 min cooked broccoli samples, and finally by the 1 min cooked samples. Thus, broccoli consumption between hyperlipidemic animal models controls BG levels similar to a previous study (Chang et al. 2011) that was again in agreement with Zang et al. (2015) who found that mice fed a high diet for 92 days with 0.15% dietary kaempferol glycoside supplementation declined fasting BG levels with enhanced insulin resistance. Additionally, another study with flavanol kaempferol dietary supplementation (0.05%) exposed significant enhancements in hyperglycemia and hyperinsulinemia with impaired glucose transport‐4 (Glut4) among mice fed a high‐fat diet (obese diabetic model; Alkhalidy et al. 2015). Thus, such MB samples are highly recommended as kaempferol could be released and help as a naturally hypolipidemic mediator that protects against CVD risks.

Regarding the collected lipid profiles levels that shown to be in good arrangements in addition with being correlated well with measured kaempferol levels as antioxidant presented early in the broccoli microwaved samples. Lipid profile improved between the used hyperlipidemia animal models especially between rats fed 5 min MB samples. The hyperlipidemia has been previously characterized by elevated levels of lipids that are a major risk factor for CVD. Lifestyle modifications especially dietary interventions and/or cooking conditions are crucial in managing hyperlipidemia, with particular interest in phytochemicals found in fruits and vegetables. Some of the previous similar studies particularly within different cooking procedures and different time points have similar effects on lipid profile; they improved and circulated the lipid profile especially increasing the good and healthy cholesterol (HDL) in contrast to decreasing the LDL levels in suggestion with the improvements within insulin sensitivity examined between hyperlipidemic and obese animal models so kaempferol presented in such conditions could protect atherosclerotic induction (Alkhalidy et al. 2015; Khalil 2017). Additionally, enhancements in hyperlipidemia and diabetes within obese mice, mainly down‐regulation of PPAR‐γ and SREBP‐1c increased lipid metabolism in order to show beneficial effects in cholesterol levels (Zang et al. 2015).

Kidney function especially urea levels where at the highest levels between rats groups that consumed 5 min MB samples while the lowest levels were seeing between rats group fed 2 min MB comparing to positive and negative control groups. Moreover, the liver functions also showed that ALT where at its peak levels between groups fed 1 and 5 min cooked broccoli samples comparing to the lowest levels of group fed broccoli samples at 2 min. While AST levels at 5 min microwaved time point were the lowest levels between rats fed 1 min of cooked broccoli samples compared to the positive and negative groups.

The histologically presented levels showed signs of renal dysfunction, including glomerular hypertrophy and tubular dilation as indicative of hyperlipidemia‐related kidney damage, while the rat groups receiving MB showed a decrease in these pathological changes, suggesting a protective effect of kaempferol on kidney function. Histological analysis of liver sections revealed differences between the control group and the group receiving MB, especially at 5 min. Also, the group receiving MB displayed reduced lipid accumulation and improved liver cell morphology, suggesting potential protective effects of kaempferol against hyperlipidemia‐induced liver damage. Such histological findings indicate that kaempferol, delivered through the consumption of MB, employs hypo‐lipid effects significantly in hyperlipidemic rats. Also, there is a good correlation for the reduction in lipid accumulation within the liver samples and functions as well, in addition to such renal protective effects as the potential of kaempferol to mitigate hyperlipidemia, protecting vital organs from associated damage. Kaempferol's antioxidant activity could protect against lipid peroxidation and oxidative stress, which contribute to the development of hyperlipidemia that showed kaempferol's anti‐inflammatory properties associated with hyperlipidemia, thus mitigating organ damage. All the collected results after the consumption of MB samples have agreed in such liver and kidney function improvements with previous work done by Zang et al. (2015). It shows that gene expression analysis of the liver improved hyperlipidemia conditions between animal models, suggesting that kaempferol consumption reduces the accumulation of adipose tissue. All the obtained results herein between hyperlipidemic models used and after dietary intervention in different cooking conditions, especially MB consumption, agree with the revealed consumption of broccoli or kaempferol supplementations in order to improve CVD risk factors that could counteract diseases by their antioxidant capacity.

Thus, the current study highlights the potential of incorporating MB into dietary interventions for managing hyperlipidemia. The findings suggest that kaempferol, a phytochemical available in broccoli, can contribute to improved lipid profiles and protect against hyperlipidemia‐induced organ damage. Further research is needed to optimize the consumption of MB and explore the long‐term benefits of kaempferol supplementation in hyperlipidemic individuals.

## 5 Conclusion

To conclude, first, the free flavonol concentration could be released and/or damaged by heat sensitivity/treatments in different vegetables, influenced by cooking methods and times that should be considered in association with different healthy conditions, as in the current study. Kaempferol levels differ between the MB samples, as shown from all collected data. However, the most effective broccoli samples were within the cooked ones at the 2 min microwaved time point, and that was followed by the samples microwaved at 5 min, while the last indicated data samples with good effects were with the microwaved samples at 1 min. Thus, the 2 min and 5 min microwaved time points are highly recommended with such plant dietary sources, especially between hyperlipidemia models. It is important to incorporate kaempferol‐rich foods into your diet in easy ways to reap the kaempferol potential health benefits, such as adding leafy greens to your salads (kale, spinach) and green smoothie consumption for having a healthy delicious dose of kaempferol in a convenient way, in addition to maximizing the potential benefits of other bioactive compounds supporting lipid management and promoting overall health. Overall, broccoli consumption between hyperlipidemic used animal models helped to control their glucose levels in addition to their lipid, kidney, and liver functions. Additionally, kaempferol in cooked broccoli with its hyperlipidemic properties should be referred to and recommended to be distributed in different communities. Therefore, many more human studies are needed.

## Author Contributions

**Asmahan A. Ali:** conceptualization (equal), data curation (equal), formal analysis (equal), investigation (equal), methodology (equal), visualization (equal). **Huda Aljumayi:** funding acquisition (equal), investigation (equal), methodology (equal), resources (equal), visualization (equal). **Thamer Aljutaily:** software (equal), validation (equal), visualization (equal), writing – review and editing (equal). **Hani A. Alfheeaid:** validation (equal), visualization (equal), writing – review and editing (equal). **Nada Bint Abdullah Al‐Zunaidy:** software (equal), validation (equal), visualization (equal), writing – review and editing (equal). **Isam A. Mohamed Ahmed:** validation (equal), visualization (equal), writing – review and editing (equal). **Belal M. Mohammed:** validation (equal), visualization (equal), writing – review and editing (equal). **Nazeha A. Khalil:** conceptualization (equal), supervision (equal), validation (equal), writing – review and editing (equal).

## Disclosure

Declaration of Generative AI and AI‐Assisted Technologies in the Writing Process: we confirm that no AI‐assisted technologies have been used in the current article.

## Ethics Statement

The authors declare that the current study was carried out at the Nutrition and Food Sciences Department, Menoufia University, Egypt after being approved by the academic professional committee under safety and well‐being conditions. The current study has been approved ethically and biologically by the scientific research ethics committee (Animal Care and Use: 28‐SREC‐08‐2023).

## Conflicts of Interest

The authors declare no conflicts of interest.

## Supporting information


**Table S1.** Supplemental material for used basal diet.

## Data Availability

The data supporting the conclusions of this article is included in the manuscript.
