# Supplementary figures and images for: The Hypolipemic Properties of Kaempferol Presented in Microwaved Cooked Broccoli Between Hyperlipidemic Rat Models
Source: Food Sci Nutr. 2025 Jul 7;13(7):e70556. doi: 10.1002/fsn3.70556 (PMC12230359; doi:10.1002/fsn3.70556)

**Table S1; supplemental material for used basal diet**


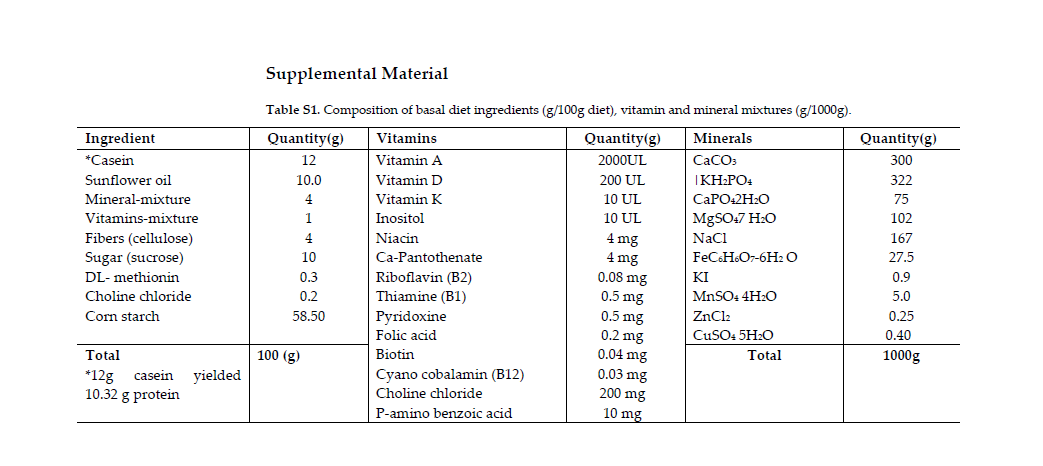

Supplement: Supplementary file 1 — Table S1. Supplemental material for used basal diet. [file FSN3-13-e70556-s001.docx]
